# Association between Fall History and Gait, Balance, Physical Activity, Depression, Fear of Falling, and Motor Capacity: A 6-Month Follow-Up Study

**DOI:** 10.3390/ijerph191710785

**Published:** 2022-08-30

**Authors:** Catherine Park, Md Moin Uddin Atique, Ramkinker Mishra, Bijan Najafi

**Affiliations:** 1 Interdisciplinary Consortium on Advanced Motion Performance (iCAMP), Michael E. DeBakey Department of Surgery, Baylor College of Medicine, Houston, TX 77030, USA; 2 VA’s Health Services Research and Development Service (HSR&D), Center for Innovations in Quality, Effectiveness, and Safety, Michael E. DeBakey VA Medical Center, Houston, TX 77030, USA; 3 Big Data Scientist Training Enhancement Program, VA Office of Research and Development, Washington, DC 20420, USA

**Keywords:** fall history, consequences of falls, wearables, older adults, gait, balance, physical activity, depression, fear of falling, motor capacity

## Abstract

Maintaining function in older adults is key to the quality of life and longevity. This study examined the potential impact of falls on accelerating further deterioration over time in gait, balance, physical activity, depression, fear of falling, and motor capacity in older adults. 163 ambulatory older adults (age = 76.5 ± 7.7 years) participated and were followed for 6 months. They were classified into fallers or non-fallers based on a history of falling within the past year. At baseline and 6 months, all participants were objectively assessed for gait, balance, and physical activity using wearable sensors. Additional assessments included psychosocial concerns (depression and fear of falling) and motor capacity (Timed Up and Go test). The fallers showed lower gait performance, less physical activity, lower depression level, higher fear of falling, and less motor capacity than non-fallers at baseline and 6-month follow-up. Results also revealed acceleration in physical activity and motor capacity decline compared to non-fallers at a 6-month follow-up. Our findings suggest that falls would accelerate deterioration in both physical activity and motor performance and highlight the need for effective therapy to reduce the consequences of falls in older adults.

## 1. Introduction

According to U.S. Centers for Disease Control and Prevention (CDC), “every second of every day, an older adult (age 65+) suffers a fall in the U.S., making falls the leading cause of injury and injury death in this age group” [1]. It has been reported that 1 in 5 falls causes a serious injury such as broken bones or a head injury, with a direct cost estimated to be exceeded $50 billion in 2015 [2,3,4]. CDC has also reported that fall death rates in the U.S. increased by 30% from 2007 to 2016 and it is expected to reach 7 falls deaths every hour by 2030 [5]. It is well established that falls affect the quality of life (sense of independence) by increasing the anxiety and fear of falling and reducing confidence in performing daily tasks [6,7,8]. However, the impact of falls on accelerating declines in functional and motor performance is overlooked.

A decline in motor performance and physical activity are major risk factors for falls in older adults [9,10,11,12]. However, few studies explored the changes in physical activities and motor performance post a fall incident in older adults, but the reported observations are often limited to self-report of physical activities, used cross-sectional study design, lack data from age-matched non-fallers, and/or had limited sample size (less than 100 subjects) [13,14,15,16,17]. Furthermore, other conditions contributing to increased risk of falls in older adults include depression, fear of falling, gender, and motor capacity [18,19]. However, no study explored the changes in psychosocial concerns (depression and fear of falling) and motor capacity in older adults.

Therefore, the main objective of this study was to quantitatively assess changes and differences in gait, balance, and physical activity in older adults with and without a history of falling in the previous year. This study also assessed other fall risk factors, including depression, fear of falling, and motor capacity. Gait and balance performance, physical activity, depression, fear of falling, and motor capacity were evaluated at baseline and a 6-month follow-up. We hypothesized that older adults with a history of falling show an accelerated deterioration in gait and balance performance, physical activity, and/or the other fall risk factors (depression, fear of falling, and/or motor capacity) compared to their counterparts without a history of falling.

## 2. Materials and Methods

### 2.1. Participants and Demographics

Participants in this study were recruited from the Baylor College of Medicine, Michael E. DeBakey Veterans Affairs Medical Center, and the University of Arizona from outpatient clinics or communities. Eligible participants were 65 years or older and could walk at least 10 m with or without walking assistance. Potential participants were excluded if they: (1) had a foot problem (wound, amputation, etc.); (2) had significant medical or psychiatric conditions; or (3) were not willing to participate.

The study protocol was approved by the local Institutional Review Boards at Baylor College of Medicine (Protocols: H-41717), Michael E. DeBakey Veterans Affairs Medical Center (Protocol: H-40765), and University of Arizona (Protocol: 12-0659-01). All participants read and signed a consent form prior to the study.

Demographics collected from all participants included age, gender, and body mass index (BMI). The following clinical information was also collected such as the number of falls in the past year, medical conditions (i.e., high blood pressure, heart disease, musculoskeletal problem, stroke, Parkinson’s disease, diabetes, osteoarthritis, rheumatoid arthritis, cancer, urinary tract problems, digestive problems, hearing problem), use of walking assistance (e.g., cane or walker), the daily number of prescribed medications, and Mini-Mental State Examination (MMSE) score.

### 2.2. Experimental Protocols

As suggested by previous literatures [20,21], we used a similar protocol to determine fallers. In summary, according to the participants’ self-reported fall history (i.e., the number of falls in the past year) at baseline, participants who reported falling at least once were assigned to the fallers, and others were assigned to the non-fallers. As recommended by the World Health Organization (WHO) [22], a fall was defined as an event which results in a person coming to rest inadvertently on the ground or floor or other lower level. All participants were assessed for their gait and balance performance, physical activity, depression, fear of falling, and motor capacity at baseline and 6-month follow-up.

**Figure 1:**
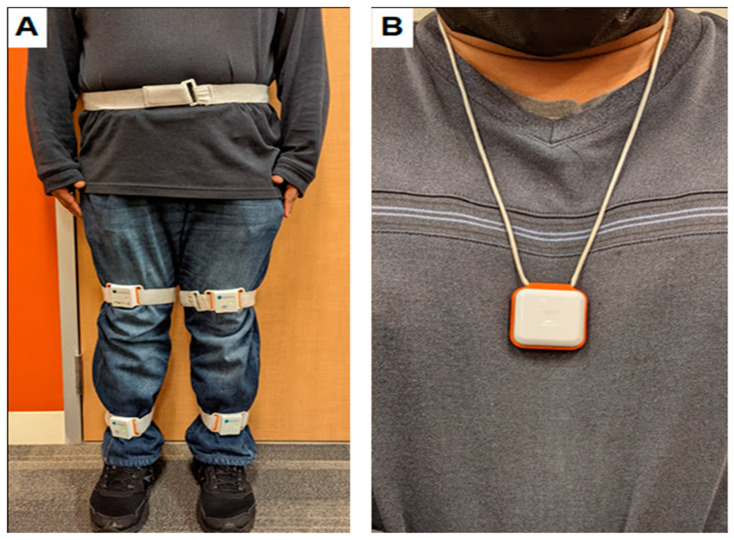
(**A**) Wearable sensors and their placement for gait and balance performance assessments. (**B**) A pendant sensor and its placement for physical activity assessments.

For gait and balance performance assessments, each participant was instrumented with a validated wearable system (i.e., LegSys™ and BalanSens™, BioSensics, Newton, MA, USA) consisting of five wearable motion sensors attached to the lower trunk, thighs, and shanks (Figure 1A). Then, all participants completed a static balance test with their eyes open and closed. During all static balance tests, participants were instructed to have their feet hip-width apart and parallel, stand upright, cross their arms at their chest, keep their knees extended, and breathe normally for 30 s [23]. After the static balance test, all participants performed single-task and dual-task walking tests for 10 m with their comfortable walking speed [24]. During single-task walking tests, participants walked without any cognitive distraction, but participants walked by counting backward from a random number during dual-task walking tests.

For physical activity assessments, all participants were asked to wear a pendant sensor (PAMSys™, BioSensics, Newton, MA, USA) at their sternum level for 48 h (i.e., two consecutive days) (Figure 1B) [25,26,27]. The 48 h duration was determined based on the results of our previous studies [25,26,27]. The PAMSys™ is small (3.5 cm (W) × 3.5 cm (H) × 1.5 cm (D)), light in weight (24 g), easy to use, and it can run for 200 h without charging.

For depression and fear of falling assessments, all participants were assessed for their depression symptoms using the Center for Epidemiologic Studies-Depression (CES-D) questionnaire [28] and their concern of falls using the Falls Efficacy Scale-International (FES-I) questionnaire [29]. The CES-D score (≥16) was used to identify participants at risk for clinical depression (i.e., possible depression) [28], and the FES-I score (≥22) was used to determine participants with a high concern of falling [29]. For motor capacity assessments, all participants completed the Timed Up and Go (TUG) test that consisted of standing up from a chair, 3-m walking, turning around, walking back and siting down [30]. Of note, we did not include dual-task TUG because a previous study has suggested that dual-task gait may better predict prospective falls [31,32].

### 2.3. Data and Statistical Analysis

Table 1 describes gait, balance, and physical activity variables. To quantify gait performance, two variables (single-task walking speed and dual-task walking speed) were calculated by validated algorithms (LegSys™ software, BioSensics, Newton, MA, USA) [33,34,35,36]. The center of mass (COM) sway area calculated by BalanSens™ software (BioSensics, Newton, MA, USA) [37,38] quantified balance performance for eyes open and closed conditions. To quantify physical activity, eight variables (% of lying, % of sitting, % of standing, % of walking, walking steps per bout, daily walking steps, duration of stand-to-sit, and duration of sit-to-stand) were calculated by PAMWare™ (BioSensics, Watertown, MA, USA) [39,40]. CES-D score, FES-I score, and TUG time were used to assess depression, fear of falling, and motor capacity, respectively.

To determine the accelerated deterioration in gait and balance performance, physical activity, depression, fear of falling, and motor capacity, we compared all variables at the outcomes at the 6-month follow-up compared to baseline between fallers and non-fallers. Measuring outcomes in two timepoints (baseline and follow-up) is considered as a usual method to determine outcomes in both clinical trials and observational studies [20,41,42,43,44].

Continuous data were expressed with mean ± standard deviation (SD). Categorical data were reported as count (%). All statistical analyses were performed using IBM SPSS Statistics version 27 (IBM Corp., Armonk, NY, USA). Shapiro-Wilk test was used to identify the normal distribution of continuous variables. A one-way ANOVA for normally distributed variables or Mann-Whitney U test for non-normally distributed variables was tested to determine to mean differences between groups (i.e., fallers and non-fallers). A chi-square test was used to test significant levels between groups for categorical variables. The effect size was calculated using Cohen’s *d* for all continuous variables associated with gait, balance, physical activity, depression, fear of falling, and motor capacity. A linear mixed model for normally distributed variables and generalized estimating equations for non-normally distributed variables was used to assess the main effects of the assessment time (baseline and 6-month follow-up) and group (fallers and non-fallers) as well as their interactions by adjusting age, gender, and BMI as potential confounders. These analyses accommodate the longitudinal design, allow for testing differences between groups in patterns over time as well as at specific time points, are consistent with an intention to treat analysis [45], and are valid for data that are missing at random [46]. To determine the accelerated deterioration in gait and balance performance, physical activity, depression, fear of falling, and motor capacity, we compared all variables at the 6-month follow-up compared to baseline between fallers and non-fallers. Measuring outcomes in two timepoints (baseline and follow-up) is considered as a usual method to determine outcomes in both clinical trials and observational studies [20,41,42,43,44]. Thus, multiple pairwise comparisons were conducted using the least significant difference method. For all statistical analyses, the significance level was set at the 2-sided *p* < 0.05.

## 3. Results

### 3.1. Demographic and Clinical Characteristics

Table 2 reports participants’ demographic and clinical characteristics at baseline for both groups, including statistical results. Statistical analysis found that gender was significant between the fallers and non-fallers. However, age and BMI were not significantly different between the groups.

Among fallers, the frequency of fall over past 12 months was on average 2.0 ± 1.2 times. Among 16 clinical characteristics, 3 clinical characteristics were significantly different between the fallers and non-fallers groups. Specifically, % of osteoarthritis, Urinary tract problems, and use of walking assistance were significantly higher for fallers than non-fallers. Notably, fallers had significantly higher likelihood of prospective falls at 6-month compared to non-fallers (57% in fallers vs. 25% in non-fallers, *p* < 0.0001).

### 3.2. Gait, Balance, and Physical Activity

Table 3 reports 12 variables associated with gait, balance, and physical activity, including statistical results. Statistical analysis showed the main effects of the group for single-task walking speed and dual-task walking speed, the main effects of the time for single-task walking speed, % of lying, walking steps per bout, and daily walking steps, and the interaction effects for walking steps per bout and daily walking steps. Statistical analysis also found that fallers had a significantly higher % of lying, fewer walking steps per bout, and fewer daily walking steps at the 6-month follow-up compared to baseline. However, the other 9 variables (single-task walking speed, dual-task walking speed, COM sway area (eyes open), COM sway area (eyes closed), % of sitting, % of standing, % of walking, duration of stand-to-sit, and duration of sit-to-stand) were not statistically different between baseline and the 6-month follow-up. The Cohen’s *d* effect size was observed as small (0.20–0.49) for 3 significant variables (% of lying, walking steps per bout, and daily walking steps).

In non-fallers, statistical analysis found that walking steps per bout were significantly fewer at the 6-month follow-up than at baseline. However, the other 11 variables (single-task walking speed, dual-task walking speed, COM sway area (eyes open), COM sway area (eyes closed), % of lying, % of sitting, % of standing, % of walking, daily walking steps, duration of stand-to-sit, and duration of sit-to-stand) were not statistically different between baseline and the 6-month follow-up. The Cohen’s *d* effect size was observed as small (0.20–0.49) for the significant variable (walking steps per bout).

At baseline, fallers had a significantly slower walking speed for two tasks (single- and dual-task) than non-fallers. However, the other 10 variables (COM sway area (eyes open), COM sway area (eyes closed), % of lying, % of sitting, % of standing, % of walking, walking steps per bout, daily walking steps, duration of stand-to-sit, and duration of sit-to-stand) were not statistically different between the fallers and non-fallers. The Cohen’s *d* effect size was observed as medium (0.50–0.79) for the dual-task walking speed and as small (0.20–0.49) for single-task walking speed.

At the 6-month follow-up, fallers had a significantly a significantly slower walking speed for two tasks (single- and dual-task), less % of standing, less daily walking steps, and longer duration of stand-to-sit than non-fallers. However, the other 7 variables (COM sway area (eyes open), COM sway area (eyes closed), % of lying, % of sitting, % of walking, walking steps per bout, and duration of sit-to-stand) were not statistically different between the fallers and non-fallers. The Cohen’s *d* effect size was observed as small (0.20–0.49) for 5 significant variables (single-task walking speed, dual-task walking speed, % of standing, daily walking steps, and duration of stand-to-sit).

### 3.3. Depression, Fear of Falling, and Motor Capacity

Table 4 reports variables associated with depression, fear of falling, and motor capacity, including statistical results. Statistical analysis showed the main effects of the group for CES-D score, FES-I score, and TUG time, the main effects of the time for FES-I score, and the interaction effects for CES-D score, FES-I score, and TUG time. Statistical analysis also found that fallers had a significantly longer time to complete the TUG test at the 6-month follow-up compared to baseline. However, the other 4 variables associated with depression and fear of falling were not statistically different between baseline and the 6-month follow-up. The Cohen’s *d* effect size was observed as very small (<0.20) for the significant variable (TUG time).

In non-fallers, statistical analysis found that FES-I scores were significantly less at the 6-month follow-up than at baseline. However, the other 4 variables (CES-D score, CES-D score ≥ 16, FES-I score ≥ 22, and TUG time) were not statistically different between baseline and the 6-month follow-up. The Cohen’s *d* effect size was observed as very small (<0.20) for the significant variable (FES-I score).

Statistical analysis found that all variables associated with depression, fear of falling, and motor capacity were significantly different between the fallers and non-fallers at baseline and the 6-month follow-up. Specifically, fallers had a higher CES-D score, a greater number of participants with a CES-D score ≥ 16, a higher FES-I score, a greater number of participants with a FES-I score ≥ 22, and a greater TUG time regardless of the assessment time (baseline and 6-month follow-up). The Cohen’s *d* effect size was observed as large (0.80–1.29) for the FES-I score at baseline and the 6-month follow-up, as medium (0.50–0.79) for the TUG time at the 6-month follow-up, and as small (0.20–0.49) for the CES-D score at baseline and the 6-month follow-up and the TUG time at baseline.

## 4. Discussion

This study examined prospective changes in gait, balance, physical activity, psychosocial concerns (depression and fear of falling), and motor capacity in older adults and compared the changes between age-matched fallers and non-fallers. To our knowledge, this is the first study exploring prospective changes in objective metrics of physical activities, gait, and balance quantified using wearables in older adults with and without a history of falls.

Our results showed accelerated deterioration in fallers for physical activities and gait. Additionally, group and time effects were observed for some physical activity and gait variables. However, no interaction (i.e., group × time) effects were observed for gait and balance variables. Among fallers, several patterns of physical activities significantly deteriorated at 6-month compared to baseline, including longer % of lying on average by 9.6%, shorter unbroken walking bout on average by 11.5%, and lower daily steps on average by 15.0%. Among non-fallers, only the average duration of the unbroken walking bout was reduced on average by 11.9%, with no significant differences for other parameters of interest. These results indicate that the fallers have accelerated deterioration in physical activities over time compared to age-matched non-fallers. These observations are in agreement with a previous study [17] in which a greater annual decline in active daily bouts and daily active minutes among fallers were reported compared to non-fallers. Compared to the previous study [17], our study, however, provides more granular details about changes over time in patterns of physical activities, including cumulative postures (changes in % of lying, sitting, and standing postures) as well as locomotion (walking bouts and step counts) among fallers and non-fallers.

Najafi et al. [39] have demonstrated that a longer duration of postural transition (sit-to-stand and stand-to-sit) indicates a higher risk of falling in older adults. Interestingly, our results suggest that, while at baseline the duration of postural transition was not significantly different between the fallers and non-fallers, at 6 months, a significantly longer duration of stand-to-sit was observed in the fallers compared to non-fallers. Shin et al. demonstrated that fallers have weaker lower limb muscle strength than non-fallers [47]. We speculated that the longer postural transition duration observed at the 6-month compared to baseline only among fallers may link to weaker lower limb muscles caused by a reduced level of physical activities, potentially because of an increase in fear of falling and depression due to experiencing a fall incident. Thus, we attribute the decreased physical activity in fallers to a decline in physical functioning and strength. Previous studies have reported that a lack of physical activity often leads to a decline in physical functioning and a higher prospective risk of falls [16,48,49]. This is aligned with our observation in which 2.28 times higher likelihood of prospective falls was observed in the fallers compared to non-fallers (57% in fallers v. 25% in non-fallers), which could be because of a further decline in physical activities among fallers.

While a deterioration trend in gait speed was observed for both groups at 6 months compared to baseline, the observed trend didn’t achieve a statistically significant level in either group in our sample. However, the gait speed was significantly lower among fallers irrespective of test conditions (single and dual-task) and time of assessments (baseline and 6 months). These results are consistent with the results of previous studies that fallers walked more slowly than non-fallers when both cohorts walked with and without dual tasks (walking while counting backward) [50,51]. At the 6-month follow-up, fallers also had a less % of standing, fewer daily walking steps, and a longer duration of stand-to-sit than non-fallers. We attribute these results to a decline in physical functioning and strength in fallers.

Our results showed accelerated deterioration in fallers for depression, fear of falling, and motor capacity. Additionally, group effects were observed for all depression, fear of falling, and motor capacity, and time effects were observed for fear of falling. Particularly, fallers had a longer TUG time at the 6-month follow-up than baseline, which could be related to a decline in physical functioning and strength. Interestingly, non-fallers had a lower FES-I score at the 6-month follow-up than baseline. This result may be due to reliance on subjective participant-reported outcomes. Notably, 25% of non-fallers fell at least once between baseline and the 6-month follow-up, which is not likely reflected in FES-I scores.

The results of group comparisons at baseline and the 6-month follow-up showed differences for all variables associated with depression, fear of falling, and motor capacity. Specifically, fallers had higher CES-D scores, higher risks for depression, higher FES-I scores, greater concerns of falling, and longer TUG time regardless of the assessment time (baseline and 6-month follow-up). These results are in agreement with the findings in previous studies that depression, fear of falling, and motor capacity are fall risk factors [18,19,52].

Consistent with previous studies, our results showed that the fallers had a higher rate of osteoarthritis [53], urinary tract problems [54], and walking assistance use [55]. Notably, fallers had a higher recurrent rate of falling than non-fallers between baseline and the 6-month follow-up. This result is not surprising because ample evidence indicates that older adults with a history of falling are more likely to experience recurrent falls within a few months [56].

Of note, the main purpose of the present study was to examine the change of gait, balance, physical activity, depression, fear of falling, and motor capacity between faller and non-faller classified self-reported falls of the past year from baseline. The limitations of this study include non-objective self-reporting outcomes (i.e., fall history, CES-D, and FES-I), an underpowered sample size to confirm some of the study’s observations, and an imbalanced gender distribution within and between the two groups. An additional limitation is a relatively short period of follow-up. To determine the accelerated deterioration in gait and physical activity outcomes among fallers, we compared the outcomes at 6 months compared to baseline between fallers and non-fallers. However, this method may limit the measurement to 1 point of data along time, which may not be sufficient to provide a clear trajectory of changes in gait and physical activity over time. The current observational study was unable to determine the importance of the measured individual risk factors at baseline on prospective falls as well as on the magnitude of deterioration in gait and physical activity at 6 months. Additionally, the duration of 6 months may not be sufficient to observe noticeable changes in some of the parameters of interest. Despite these limitations, our study confirms the need for timely intervention to improve physical activities among fallers, which may help preserve functional and motor performance and consequently prevent future falls. Our future studies will increase the same size, match gender distribution, adopt multiple measurements, and use an extended duration of follow-up (e.g., more than one year or a few years). Additionally, we will use the machine learning technique to identify dominant variables affected by fall history in the past year and predict a future fall.

## 5. Conclusions

In a 6-month prospective observational study using objective monitoring of physical activities, gait, and balance between two age-matched cohorts of older adults with and without a history of falls, we observed accelerated deterioration in patterns of physical activities among fallers compared to non-fallers. The deteriorations in the level of physical activities among fallers were more pronounced for shorter walking bouts, lower daily steps, and longer % of lying postures. Additionally, the duration of stand-to-sit postural transition was significantly longer among fallers at 6 months compared to non-fallers, indicating a higher risk of fallings. These observations highlight the need for an immediate intervention post a fall incident to limit the consequences of falls, particularly preventing further deterioration in physical activity patterns, which may lead to higher risk or prospective falls. However, our observations need to be confirmed in a larger sample and over an extended follow-up period.

## Figures and Tables

**Table 1:** Description of gait, balance, and physical activity variables.

Name	Unit	Description
Single-task walking speed	m/s	Average walking speed for 10 m without cognitive tasks
Dual-task walking speed	m/s	Average walking speed for 10 m with cognitive tasks
COM sway area (eyes open)	cm^2^	95% of eliptical area of COM sway with eyes open
COM sway area (eyes closed)	cm^2^	95% of eliptical area of COM sway with eyes closed
% of lying	%	Percentage of lying time for 24 h
% of sitting	%	Percentage of sitting time for 24 h
% of standing	%	Percentage of standing time for 24 h
% of walking	%	Percentage of walking time for 24 h
Walking steps per bout	n	Average of daily number of steps per unbroken walking
Daily walking steps	n	Daily number of total walking steps
Duration of stand-to-sit	s	Duration of stand-to-sit transitions for 24 h
Duration of sit-to-stand	s	Duration of sit-to-stand transitions for 24 h

**Table 2:** Demographic and clinical characteristics at baseline for the fallers and non-fallers groups.

	Participants, No. /Total No. (%), by Group		*p*-Value
	Fallers (*n* = 65)	Non-Fallers (*n* = 98)	
Demographics			
Age, years	77.5 ± 8.6	76.8 ± 8.0	0.656
Gender (Female), *n* (%)	54 (83.1)	60 (61.2)	0.003 *
Body Mass Index, kg/m^2^	26.8 ± 8.6	25.8 ± 8.5	0.462
Clinical Characteristics			
Number of falls in past year, *n*	2.0 ± 1.2	-	-
High blood pressure, *n* (%)	38/60 (63.3)	48/90 (53.3)	0.225
Heart disease, *n* (%)	13/60 (21.7)	17/90 (18.9)	0.677
Musculoskeletal problem, *n* (%)	22/60 (36.7)	36/90 (40.0)	0.681
Stroke, *n* (%)	5/60 (8.3)	4/90 (4.4)	0.326
Parkinson’s disease, *n* (%)	0/60 (0.0)	1/90 (1.1)	0.413
Diabetes, *n* (%)	16/64 (25.0)	18/97 (18.6)	0.327
Osteoarthritis, *n* (%)	28/60 (46.7)	24/90 (26.7)	0.012 *
Rheumatoid Arthritis, *n* (%)	7/60 (11.7)	4/90 (4.4)	0.096
Cancer, *n* (%)	15/60 (25.0)	22/90 (24.4)	0.938
Urinary tract problems, *n* (%)	20/60 (33.3)	16/90 (17.8)	0.029 *
Digestive problems, *n* (%)	11/60 (18.3)	18/90 (20.0)	0.800
Hearing problem, *n* (%)	23/60 (38.3)	29/90 (32.2)	0.441
Walking assistance use, *n* (%)	28/61 (45.9)	18/89 (20.2)	0.001 *
Number of prescription medications	4.7 ± 3.7	3.9 ± 2.9	0.224
Number of over the counter medications	2.7 ± 4.1	2.7 ± 2.5	0.368
Mini-mental State Exam score	28.4 ± 2.1	28.2 ± 2.6	0.633

Values are presented as mean ± standard deviation or *n* (%). Asterisk denotes a significant difference between the groups.

**Table 3:** Main and interaction effects and comparison of gait, balance, and physical activity variables as a function of the assessment time (baseline and 6-month) and group (fallers and non-fallers).

	Main and Interaction	Fallers (*n* = 65)			Non-Fallers (*n* = 98)			Fallers vs. Non-Fallers	
	Group (G) *p*-Value Time (T) *p*-Value G × T *p*-Value	Baseline	6-Month	*p*-Value (*d*-Value)	Baseline	6-Month	*p*-Value (*d*-Value)	Baseline *p*-Value (*d*-Value)	6-Month *p*-Value (*d*-Value)
Gait									
Single-task walking speed, m/s	G = 0.006 * T = 0.037 * G × T = 0.830	0.92 ± 0.32	0.89 ± 0.28	0.088 (0.10)	1.04 ± 0.24	1.02 ± 0.26	0.166 (0.08)	0.010 * (0.44)	0.007 * (0.48)
Dual-task walking speed, m/s	G = 0.003 * T = 0.371 G × T **=** 0.515	0.78 ± 0.30	0.81 ± 0.31	0.285 (0.10)	0.94 ± 0.25	0.94 ± 0.30	0.850 (0.00)	0.001 * (0.59)	0.011 * (0.43)
Balance									
COM sway area (eyes open), cm^2^	G = 0.768 T = 0.146 G × T **=** 0.523	0.46 ± 0.41	0.51 ± 0.48	0.515 (0.11)	0.45 ± 0.45	0.57 ± 0.89	0.188 (0.17)	0.814 (0.02)	0.584 (0.08)
COM sway area (eyes closed), cm^2^	G = 0.226 T = 0.224 G × T **=** 0.490	1.11 ± 1.57	0.98 ± 0.82	0.516 (0.10)	1.61 ± 4.12	1.23 ± 1.78	0.303 (0.12)	0.298 (0.15)	0.255 (0.17)
Physical Activity (Cumulated Posture Duration)									
Lying, *%*	G = 0.818 T = 0.045 * G × T **=** 0.133	39.5 ± 11.8	43.3 ± 10.9	0.026 * (0.33)	40.8 ± 12.2	41.3 ± 11.2	0.732 (0.04)	0.540 (0.11)	0.282 (0.18)
Sitting, %	G = 0.349 T = 0.156 G × T **=** 0.440	39.8 ± 11.5	37.5 ± 10.9	0.186 (0.21)	37.4 ± 12.2	36.7 ± 10.1	0.585 (0.06)	0.249 (0.20)	0.669 (0.08)
Standing, *%*	G = 0.128 T = 0.535 G × T **=** 0.150	15.0 ± 5.2	14.0 ± 5.0	0.132 (0.20)	15.6 ± 5.4	16.0 ± 5.5	0.550 (0.07)	0.526 (0.11)	0.042 * (0.38)
Walking, *%*	G = 0.156 T = 0.109 G × T **=** 0.143	5.7 ± 3.4	5.1 ± 3.2	0.095 (0.18)	6.2 ± 3.0	6.1 ± 3.2	0.593 (0.03)	0.369 (0.16)	0.096 (0.31)
Physical Activity (Daily Walking Performance)									
Walking steps per bout, n	G = 0.827 T = 0.001 * G × T **=** 0.011 *	30.5 ± 17.7	27.0 ± 13.7	0.032 * (0.22)	31.2 ± 16.5	27.5 ± 13.0	0.011 * (0.25)	0.837 (0.04)	0.843 (0.04)
Daily walking steps, n	G = 0.092 T = 0.039 * G × T **=** 0.015 *	3648.1 ± 2889.4	3104.3 ± 2153.0	0.045 * (0.21)	4202.0 ± 2715.8	3985.6 ± 2712.3	0.386 (0.08)	0.268 (0.20)	0.038 * (0.35)
Physical Activity (Postural Transition)									
Duration of stand-to-sit, *s*	G = 0.136 T = 0.566 G × T **=** 0.113	4.40 ± 0.89	4.49 ± 0.96	0.401 (0.10)	4.30 ± 1.15	4.13 ± 0.89	0.136 (0.17)	0.578 (0.09)	0.030 * (0.39)
Duration of sit-to-stand, *s*	G = 0.169 T = 0.904 G × T **=** 0.279	4.34 ± 1.00	4.46 ± 1.25	0.526 (0.11)	4.27 ± 1.10	4.12 ± 0.88	0.256 (0.15)	0.702 (0.07)	0.080 (0.33)

Values are presented as mean ± standard deviation or *n* (%). *d* indicates Cohen’s *d* statistic effect size. Asterisk denotes a significant difference between the groups. Results were adjusted by age, gender, and body mass index.

**Table 4:** Main and interaction effects and comparison of depression, fear of falling, and motor capacity variables as a function of the assessment time (baseline and 6-month) and group (fallers and non-fallers).

	Main and Interaction	Fallers (*n* = 65)			Non-Fallers (*n* = 98)			Fallers vs. Non-Fallers	
	Group (G) *p*-Value Time (T) *p*-Value G × T *p*-Value	Baseline	6-Month	*p*-Value (*d*-Value)	Baseline	6-Month	*p*-Value (*d*-Value)	Baseline *p*-Value (*d*-Value)	6-Month *p*-Value (*d*-Value)
Depression									
CES-D, score	G = 0.004 * T = 0.064 G × T **=** 0.010 *	8.5 ± 6.9	10.1 ± 7.7	0.085 (0.22)	6.3 ± 5.9	6.8 ± 6.4	0.435 (0.08)	0.034 * (0.35)	0.005 * (0.48)
CES-D score ≥ 16, *n* (%)		13/64 (20.3)	12/64 (18.8)	0.824	8/96 (8.3)	7/96 (7.3)	0.788	0.028 *	0.028 *
Fear of Falling									
FES-I, score	G < 0.0001 * T = 0.017 * G × T < 0.0001 *	32.3 ± 12.5	30.8 ± 12.1	0.144 (0.12)	24.1 ± 8.1	22.7 ± 8.5	0.038 * (0.17)	<0.0001 * (0.81)	<0.0001 * (0.80)
FES-I score ≥ 22, *n* (%)		46/64 (71.9)	42/64 (65.6)	0.446	39/95 (41.1)	29/95 (30.5)	0.130	<0.0001 *	<0.0001 *
Motor Capacity									
TUG time, s	G = 0.006 * T = 0.136 G × T **=** 0.048 *	14.9 ± 7.6	16.2 ± 10.5	0.043 * (0.14)	12.0 ± 4.7	12.0 ± 6.5	0.901 (0.00)	0.010 * (0.48)	0.007 * (0.50)

Values are presented as mean ± standard deviation or n (%). *d* indicates Cohen’s *d* statistic effect size. Asterisk denotes a significant difference between the groups. Results were adjusted by age, gender, and body mass index.

## Data Availability

The datasets are available upon request to the corresponding author.
